# Latest Advances in Sow Nutrition during Early Gestation

**DOI:** 10.3390/ani11061720

**Published:** 2021-06-09

**Authors:** Pieter Langendijk

**Affiliations:** Trouw Nutrition Innovation, 3811 MH Amersfoort, The Netherlands; pieter.langendijk@trouwnutrition.com

**Keywords:** nutrition, pigs, gestation, embryos

## Abstract

**Simple Summary:**

The first month of gestation in the pig is characterised by many changes, such as entry of the embryos into the uterus, increased secretion of the pregnancy hormone, progesterone, and the implantation of the embryos into the uterus. Before implantation, embryos have not yet attached to the uterus, and are reliant on nutrients and signals in the uterine fluids, which are controlled by progesterone, among others. High feed intake has a positive effect on progesterone secretion and supports the establishment and development of pregnancy. Very low feed intakes, such as in sows competing for feed in group housing, can be detrimental to embryo survival and even the maintenance of pregnancy. After implantation, specific nutrients that improve the vascularisation of the placenta, such as arginine, can support the capacity of the uterus to support a litter of developing embryos since in this stage competition between embryos starts to play a role.

**Abstract:**

In the pig, the establishment and maintenance of luteal function in early gestation is crucial to endometrial function, embryo development, and survival. The level of feed intake has a positive effect on formation of luteal tissue and progesterone secretion by the ovaries in the pre-implantation period, which is important for endometrial remodeling and secretion. These effects are independent of luteinising hormone (LH) and probably driven by metabolic cues, such as insulin and insulin-like growth factor (IGF-1), and seem to support progesterone secretion and delivery to the endometrium, the latter which occurs directly, bypassing the systemic circulation. Even after implantation, a high feed intake seems to improve embryo survival and the maintenance of pregnancy. In this stage, luteal function is LH-dependent, although normal variations in energy intake may not result in pregnancy failure, but may contribute to nutrient supply to the embryos, since in this phase uterine capacity becomes limiting. Feed incidents, however, such as unintended fasting of animals or severe competition for feed, may result in embryo or even pregnancy loss, especially in periods of seasonal infertility. Specific nutrients such as arginine have a role in the vascularisation of the placenta and can improve the uterine capacity in the period after implantation.

## 1. Introduction

The first 30 days of gestation are critical to the success of pregnancy in pigs. In this period, pregnancy is either established successfully or, when there is insufficient interaction between embryos and the uterus, the pregnancy is lost, or embryo survival is compromised. In this same period, the potential litter size is established, determined by the number of embryos that survive. This paper describes the effects of nutrition on the establishment of pregnancy and the survival and development of embryos in pigs. The aim of this paper is to describe the complexity of events during early gestation and to explain how nutrition management can take into account the effects on luteal development and embryo survival.

## 2. Focus of Nutrition: Establishment of Pregnancy, Embryo Survival, and Embryo Development

During the first month of gestation there are a number of processes and events that have a major impact on reproductive performance. Luteal tissue formation is critical to establish pregnancy and important to ensure an optimal uterine environment for the development, maintenance, and survival of embryos. At some stage, embryos start migrating, elongating, and implanting in the uterine horns, and during these developmental phases, embryos signal to the uterus. The communication between embryos and uterus is commonly referred to as ‘maternal recognition’, and ensures the maintenance of luteal tissue, sustained progesterone secretion, and pregnancy. As the litter of embryos goes through the various stages of development and implantation, a percentage will be lost due to embryo-intrinsic factors, the uterine environment, or to differences between embryos in development. These processes will be outlined briefly below, and in the second part of this paper, the interaction with nutritional effects will be examined.

### 2.1. Luteal Tissue Formation and Maintenance

The basis of a successful pregnancy and maximal embryo survival is the formation of ovarian luteal tissue, a process which is triggered by the preovulatory LH (luteinising hormone) surge causing luteinisation of the dominant follicles. In contrast to other species, porcine corpora lutea are maintained and remain critical for the whole duration of pregnancy, and luteolysis at any stage of pregnancy will result in loss of pregnancy. Porcine luteal tissue grows rapidly and gains maximum size between day 10 and 12 after ovulation, with a total luteal mass around 6 to 8 g in gilts and 10 to 15 g in multiparous sows [1]. The total luteal mass is correlated to the number of follicles that ovulate (correlation coefficient between 0.45 and 0.62; [2,3,4]), and therefore, older sows have more luteal tissue than gilts and primiparous sows. Embryos start secreting oestrogens at ~12 days, a signal for maternal recognition that ensures that luteolysis does not occur when it would normally occur in non-pregnant animals (day 14–15), and in doing so, maintain luteal function. If luteal tissue is maintained beyond day 12, the amount of tissue remains fairly stable during the remainder of the embryonic phase.

### 2.2. Progesterone, Prostaglandins, and Remodeling of the Endometrium

During the first month of pregnancy, systemic progesterone roughly follows the growth of the luteal tissue, increasing linearly until around day 12 after ovulation, then dropping slightly and levelling for the remainder of the embryonic phase [1]. The close match between the dynamics of the luteal tissue and progesterone underlines the importance of sufficient luteal tissue for establishment of pregnancy and for the control of the uterine environment.

Clearly, progesterone is important for the remodeling of the endometrial lining in order to facilitate the implantation and delivery of nutrients to the embryos. This is reflected in the correlation between embryo survival to day 35 (r = 0.48 [5]; r = 0.72 [6]) and systemic progesterone in the first three days of gestation. Beyond these first three days, progesterone does not seem to be correlated to embryo survival [3,7], suggesting that the level of progesterone is mainly limiting in the phase where it is still on the rise. At later stages progesterone receptors in the uterus seem to drop [8,9], which may indicate that the role of progesterone changes. Alternatively, in later stages, the level of progesterone may no longer be limiting.

Embryos signal their presence through the secretion of oestrogens, and in addition, through the secretion of PGE (prostaglandin E2). These factors redirect PGF (prostaglandin F2α) secretion away from the ovaries (where PGF would exert a luteolytic effect in the absence of pregnancy) and, in addition, render the luteal tissue less sensitive to PGF. In addition to these antiluteolytic effects, oestrogens and PGE also stimulate PGE secretion by the endometrium, and together with progesterone, initiate the remodling of the endometrium. These processes were comprehensively reviewed by Waclawik et al. [9], who also suggested that PGF has a dual role, switching from a luteolytic role in the absence of pregnancy to supporting the remodeling process in the endometrium in the presence of pregnancy. The combined effects of progesterone, PGFs, and PGEs result in a complex of inflammatory-like processes in the endometrium, involving growth factors such as VEGFs (vaso-endothelial growth factor), IGFs (insulin-like growth factor), TGFs (transforming growth factor), and EGFs (epidermal growth factor), which are involved in glandular formation, histotroph secretion, and angiogenesis [9]. The potential of nutritional interventions in these specific pathways have yet to be investigated.

### 2.3. Embryo Elongation, Maternal Recognition, and Embryo Survival

The majority of prenatal losses in pigs occur during the embryonic phase (before day 35), with 20 to 30% of embryos lost by day 21, and another 10 to 15% lost by day 35 [10]. It is generally assumed that in the case of proper oestrus detection and insemination conditions, close to 100% of oocytes are fertilised, and the resulting embryos will develop and reach the uterus by around day 5–6 after fertilisation.

Between day 8 and day 10, embryos, which are still ovoid at this stage, spread through the uterine horns where some will cross the bifurcation between the left and right horn. Between days 10 and 13, embryos elongate and space themselves along the uterine horns. At this stage, most embryos still survive (93–96%, [11]), and as they start secreting oestrogens and spacing, the embryos position themselves throughout the uterine horns, aligning themselves in preparation for implantation at around day 15 [12]. The process of spacing is a coordinated mechanism, during which the available uterine space is distributed fairly evenly among the embryos. Studies with inert, oestrogen-soaked beads have shown that this process may be facilitated by mild contractile activity of the uterus, induced by oestrogens originating from the embryos, with the presence of more or less embryos in specific areas of the uterus inducing more or less uterine contractility [13]. Spacing is independent of the number of embryos, and both small and large litters migrate and space evenly [14]. Whether there is direct communication between the embryos is not clear. From our own observations, implantation sites hardly ever overlap, which would suggest that there is. In the process of spacing and implantation, the developmental stage of the embryos and the variation between the embryos play a role in determining which embryos survive [15].

Before implantation, the variation in embryonic development may be such that advanced embryos influence the uterine environment in a way that is detrimental to more delayed embryos. Geisert et al. [16] showed that the treatment of sows with oestrogens before day 12 had a negative impact on embryonic survival. Embryos will still elongate, but do not survive to day 16 [17]. This suggests that there is a window before elongation, in which oestrogens are detrimental to embryonic development through their effect on the uterine environment, relative to the developmental stage of the embryos. Treatment with oestrogens at a later stage, when embryos start to secrete oestrogens themselves, does not appear harmful to embryonic survival [13]. Advanced embryos may start to secrete oestrogens at a stage when underdeveloped embryos are compromised by the same oestrogens, and this may be another mechanism through which variation in embryonic development causes embryonic mortality.

Experimental modifications of the uterine space per embryo by using superovulation [14], superinduction [18], ligation of uterine horns [19], hemi hystero-ovariectomy [20], and unilateral oviduct ligation [21] has shown that uterine space limits the survival of embryos to day 35 of gestation, hence the term uterine capacity. The timing of embryo loss, and what limitations or factors drive these losses in different stages is important, however, only few studies provide information on these aspects. Some older studies in gilts suggest that 18 to 35% of embryos are lost to day 25, that these losses are mostly independent of space [12,22,23], and that uterine space only becomes limiting for survival after day 25.

More recent evidence in modern genotypes [24] demonstrates that around 40% of the embryos do not survive to day 35, and that two-thirds of the losses occur before day 21 in multiparous sows ovulating over 20 oocytes. By including a group of sows in which unilateral oviduct ligation was performed to provide more space per embryo, it was demonstrated that the losses before day 21 were unaltered and independent of space (Table 1). In both the intact sows and in the unilateral oviduct ligation model, 25% of embryos were lost by day 21. However, there were no embryo losses after day 21 in the unilateral oviduct ligation model, where embryos had ample space, whereas another 17% of embryos were lost after day 21 in the intact sows. Interestingly, the effect of uterine space was also evident from the length of the implantation sites and the embryo weight, which were 22% and 14% greater in the unilateral oviduct ligation model compared to the intact sows. This suggests that at or after implantation, competition between embryos for space results in mortality or reduced development of those embryos with insufficient space.

Collectively, this demonstrates that most (2/3) embryonic losses occur between days 12 and 21, around the time of implantation, and are not related to uterine capacity, but probably more to the variation between embryos in development as described above. One-third of embryo losses occur after day 21 and appear to be driven by the limitations of uterine capacity. These aspects need to be taken into account when considering the effects of nutrition on embryo survival and development, meaning that losses before and around implantation should be addressed by nutritional strategies that target the variation in embryo development, whereas losses after implantation should be addressed by nutritional strategies that target uterine capacity and functional space. This also means that nutritional strategies may require to differentiate between different windows of gestation, an approach that becomes feasible with today’s precision feeding technology.

## 3. The Role of Nutrition in Luteal Tissue Formation, Progesterone, and Embryo Development Before Implantation

In pigs, apart from the LH surge that triggers ovulation, the growth of luteal tissue and the secretion of progesterone occur independent of LH, at least until 10–12 days after ovulation [25]. Effects of increased energy intake on LH that have been reported for other reproductive processes, such as for follicle development and the number of ovulations (e.g., [26]), would therefore not apply to luteal development in this early period of pregnancy. However, the formation of luteal tissue in this early period of the embryonic phase is affected by nutrition, both before and after ovulation.

The effects of nutrition before ovulation are evident from studies where feed restriction in the preceding luteal phase and follicular phase was applied to manipulate follicular dynamics (flushing). These studies were based on the principle that during periods of low energy intake or negative energy balance, endocrine cues that stimulate follicle development, such as FSH (follicle stimulating hormone) and LH, are suppressed directly at the hypothalamus–pituitary level, or indirectly through the effects of glucose and insulin regulation signaling at the ovarian level. In short, during the follicular phase, when late antral follicles are selected to become the dominant and ovulatory pool of follicles (e.g., [26]), but also earlier during the luteal phase in gilts, or during lactation in weaned sows (e.g., [6]). When early antral follicles are recruited to develop into late antral follicles, restricted energy intake suppresses follicle development with lower ovulation rates as a result.

In more recent studies, feed restriction in cyclic gilts during the preceding luteal or follicular phase has been shown to have negative carry over effects on the rise in peripheral progesterone after ovulation [2,27], with some of these effects being due to reduced formation of luteal tissue [28]. Similarly, feed restriction in primiparous sows during the lactation preceding post-weaning ovulation, reduces post-ovulatory luteal function and progesterone [29]. The administration of insulin can counteract these effects in vivo [2] and results in increased progesterone secretion in vitro [30], suggesting that insulinogenic diets would support the formation of luteal tissue and the secretion of progesterone. In that respect, it is interesting to note that lactation diets rich in fast carbohydrates (starch and sugar) increased peripheral progesterone post ovulation [31], and that in post-weaning multiparous sows, pre-ovulatory blood insulin levels were correlated with post-ovulatory progesterone [32].

These examples illustrate how energy intake and insulinogenic ingredients that affect follicular dynamics, may benefit post-ovulatory luteal development and function. This may be through carry over effects of pre-ovulatory diets on the quality of the luteal tissue, and through effects on the amount of luteal tissue due to an increased ovulation rate.

Nutritional interventions during the post-ovulatory period can also affect the formation of luteal tissue and progesterone secretion (Table 2). In both gilt and multiparous sow models, feed restriction during early gestation reduced the amount of luteal tissue at day 30–35 of gestation [3,7], but also as early as day 10 after ovulation [33]. Considering that up to day 10–12 of pregnancy the formation of corpora lutea is LH-independent, the effects of nutrition in this early period are more likely to be mediated by other factors. Similar to premating nutrition, post-mating effects on luteal function may be mediated by insulin or insulin-related pathways such as IGF-1. Both insulin and IGF-1 are higher in gilts fed at a high feeding level in early pregnancy [34,35]. IGF-1 stimulates progesterone secretion by luteal tissue in vitro [36] and in vivo [37] and supports luteal tissue formation [38]. In primiparous sows, Langendijk et al. [39] reported a correlation between IGF-1 and post-ovulatory progesterone. Collectively, these data point to a role of feed or energy intake in growth of luteal tissue and the secretion of progesterone, and would suggest that insulin and IGF-1 stimulating dietary strategies would enhance luteal development in early pregnancy.

It is evident from the above that increased energy or feed intake during luteal tissue formation increases the secretory capacity of the ovaries. Nevertheless, from studies in the ’90s it would appear that a high feed allowance may be detrimental to embryo survival. This was mainly based on observations in gilts that a high feed level reduces systemic concentrations of progesterone due to a faster breakdown in the liver [40], and in a study by Jindal et al. [41], who showed that embryo survival was reduced when gilts were fed a high allowance in the first 3 days after ovulation, presumably due to reduced systemic progesterone. However, the results from other studies comparing feed levels throughout the whole embryonic period are equivocal, which may sometimes be due to misinterpretation of results. As reviewed by Langendijk [42], studies where embryos were recovered before implantation may have inaccurately assessed the effects of energy intake on embryo survival, since in this period the fragility of the embryos and morphological aspects once embryos start to elongate can complicate the assessment of the number of embryos (e.g., [28]). Most studies where embryos were recovered after implantation, do not report reduced embryo survival at a high energy intakes, and even reported positive effects on pregnancy rate and embryo survival (see for review [42]). A recent meta-analysis by Leal et al. [43] summarizing the effect of post-insemination energy intake on embryonic survival, echoed these observations, in that a high feed intake during the embryonic phase is not detrimental at all and is more likely beneficial to embryo development. The apparent parodox between the (negative) effects of energy intake on systemic progesterone and the (positive) effects on embryo survival, is due to the differential effects of feed level at the utero–ovarian level and at the systemic level, which will be explained below.

## 4. Ovarian and Systemic Progesterone Dynamics

Progesterone delivered to the uterus is a sum of progesterone in the systemic circulation, which is subject to hepatic clearance, and a direct transfer from the ovaries that is not subject to hepatic breakdown. Direct transfer from the ovaries is a combination of countercurrent transfer and shunts between ovarian veins and uterine arteries [1]. Therefore, the effects of nutrition on the delivery of progesterone to the uterus are determined by the net result of effects on systemic progesterone and ovarian secretion of progesterone. The direct supply of progesterone forms a significant contribution to the uterine supply since the unilateral removal of one ovary reduces embryo survival in the ipsilateral horn even though initial embryo distribution over the two horns remains unaltered [44]. Due to the direct transfer from the ovaries, progesterone in uterine arteries is much higher than in systemic circulation [45]. Since concentrations of progesterone in the local circulation around ovaries and uterus is not subject to breakdown by the liver, it is not affected by feeding plane effects on systemic progesterone.

To illustrate this, progesterone can be measured in the vena cava, at the site where the utero-ovarian vein empties into the vena cava [33,46]. At day 6 after ovulation, progesterone secretion by the ovaries followed a pulsatile pattern with 7.2 pulses per 12 h on average, as opposed to a non-pulsatile, constant concentration in the systemic concentration. The mean concentration in the vena cava at this site was much greater than in systemic circulation (88 ng/mL vs 19.6 ng/mL), and the mean amplitude of the progesterone pulses was 173 ng/mL [33]. Gilts that were fed a high feed level (2.8 vs 1.5 kg/d) had more progesterone pulses, a higher mean progesterone, and greater pulse amplitudes in the vena cava. In the same period, progesterone in the systemic circulation was reduced at the high feed level. At day 9 of pregnancy, similar observations were made. Interestingly, gilts on a higher feed allowance had improved embryo survival as early as day 10 of pregnancy (92% vs. 77%), at a stage when embryos are ovoid and recovery can be performed more accurately.

Therefore, an increased feed intake may increase ovarian progesterone secretion and direct transfer to the uterus, whereas at the same time, systemic progesterone is reduced by a higher hepatic breakdown. The net result of these divergent effects will determine the delivery of progesterone to the uterus, and may result in reduced systemic progesterone, whilst at the same time progesterone delivery to the uterus is increased. Just after ovulation, when the amount of luteal tissue is still small, the contribution of systemic progesterone may still be relevant, and this would explain the negative effect of feed level in the first 3 days post ovulation in the study by Jindal et al. [41], as opposed to the positive effect in the period thereafter. Once luteal tissue has attained a certain mass, a direct transfer of ovarian progesterone is probably far more important than small variations in systemic progesterone.

Increased ovarian progesterone secretion when energy intake is increased, and local transfer of that progesterone to the uterus, would therefore benefit the remodeling and the secretory function of the uterus leading up to implantation, and improve embryo survival before and after implantation. The beneficial effects of energy intake on luteal function and embryo survival have been reviewed by [1,42], and more recently by [43].

## 5. Specific Nutritional Effects in the Pre-Implantation Phase

Most of the research on nutrition in the early gestation in pigs has focused on the feed level paradigm. There are far fewer studies on the specific nutrients that target specific events during embryo development and attachment. Considering that in the pre-implantation phase embryos cannot rely on nutrient supply through the placenta, nutritional and developmental cues have to be transferred through uterine fluids secreted by the endometrial glands (histotroph). Changes in the concentrations of leucine, arginine, glutamine, glucose, and fructose in uterine and trophoblast fluids in the period of blastocyst development and trophoblast elongation [47] suggest that these molecules may act as functional nutrients for early embryo development. These findings have been confirmed by in vitro studies [47]. The secretion of these functional nutrients and the expression of their transporters are regulated by progesterone and oestradiol. Whether dietary intake of these nutrients limits embryo development is not known.

One vitamin that has received more attention is folic acid. Based on a review by Lindemann et al. [48] supplementation of gestating sow diets with folic acid improves litter size, but how exactly remains unclear. Most commercial diets will add folic acid at 1 to 15 mg/kg [49], but how important folic acid is for the embryo development and whether reported effects are due to deficiencies or due to supranutritional effects is not clear. Supplementation with folic acid may influence the uterine environment early on since Matte et al. [50] reported increased PGE2 in uterine flushings at day 12 and day 15 of gestation, a higher total protein content of embryos, and increased in vitro oestrogen secretion of embryonic cells from sows supplemented with 15 mg/kg folic acid. There was no evidence reported on the number of embryos or their morphological development. Folic acid in the unsupplemented diet was probably somewhere between 0.2 to 0.8 mg/kg, which is well below a standard commercial diet. In sows that had a high ovulation rate following eCG treatment, folic acid supplementation seemed to increase embryo survival [51], however, other studies by the same group [52,53] did not repeat this effect. Most likely, the effects of folic acid supplementation on embryo development are only evident when the control diet is insufficient.

An interesting vitamin that has received very little attention is riboflavin (vitamin B2). This vitamin appears to be increased in uterine flushings in a specific window from day 7 to 9 after conception. When sows were supplemented with 100 mg/d instead of the recommended 6 mg/d between days 4 and 10 of gestation, pregnancy rates and embryo survival to day 30 of pregnancy was increased [54]. The mechanism underlying this effect, however, is not clear and to our knowledge there have been no further reports on riboflavin in the literature in relation to embryo development.

## 6. Effects of Nutrition from Implantation Onwards

Beyond days 10–12 of gestation, luteal tissue becomes dependent on LH. Nevertheless, only a severe and chronic reduction in LH support will result in luteal regression. In most studies, only the complete inhibition of LH for 3 to 5 days will cause luteolysis and ultimately, pregnancy failure [1]. Nevertheless, alterations in LH because of feed intake may affect progesterone secretion and therefore modify the uterine environment to be more or less beneficial to the development and survival of embryos. This may affect vital cues for embryos as they are still spacing and implanting but may also affect angiogenic processes and the supply of nutrients to individual embryos once they have implanted and the placenta is developing. One could argue that at this stage the nutrient requirements of the embryo are still so low that competition would hardly, or not, affect their development. However, altering nutrient supply by altering the competition between embryos, such as in the unilateral oviduct ligation model [24], has demonstrated that even in the fourth and fifth weeks of pregnancy, limiting the nutrient supply or available space can significantly limit embryo size and increase embryo mortality. This illustrates the importance of optimizing the uterine environment around implantation and facilitating the process of implantation and angiogenesis.

Another example of how feed intake, or better, feed incidents, may affect embryo development is from a study where gilts were fasted completely on days 10 and 11 after conception. Fasting did not affect LH secretion or pulsatile secretion during the actual fasting. However, fasting did reduce systemic progesterone in the days following fasting, and reduced the number of piglets born at term by two [55]. This would suggest that endocrine alterations around the time of elongation and implantation, caused by feed incidents prior to that can affect embryo development and survival. Similar effects were reported by Kongsted et al. [56], who observed that in group-housed, floor fed sows, those that lost back fat in the first month of gestation had a higher risk of losing pregnancy. Some of the effects of group housing on embryo mortality and loss of pregnancy that are attributed to social stressors, therefore, may in reality be nutritional effects. High fibre diets have been used in group housing systems to increase satiety and as such reduce hunger-related stress. Isocaloric inclusion of 10% crude fibre in early gestation diets resulted in farrowing rates and litter size similar to the control diet in first-litter sows [57] and in gilts [58], and did not affect the embryo survival or pregnancy rate [3]. Fibre inclusion, however, may have to be high enough to allow close to ad lib feeding to achieve sufficient satiety [59,60]. Therefore, fibre may be a nutritional management asset, but must be high enough and aligned with measures to ensure sufficient energy intake in all sows.

Of specific functional nutrients that influence post implantation development, arginine has been studied by far the most over the last few decades. Arginine is a vasoactive and promotes blood perfusion directly through vasodilation and indirectly by stimulating angiogenesis. Both processes are mediated through the arginine-induced production of nitric oxides [61,62]. Blood perfusion has been proposed to improve placenta function and hence increase uterine capacity. When supplemental arginine (20–25 g/day) was fed from day 14 to 28 [63], or from day 30 of gestation until farrowing [64], litter size was increased. In superovulated gilts, supplementing arginine (40 g/d) between day 16 and 28 of gestation increased embryo survival at day 30 of gestation [62]. In another study with multiparous sows [61], supplemental arginine from day 15 to 29 of gestation increased foetal weight at day 49 of gestation by 6 %, although foetal survival was not changed. The studies with arginine not only show that this amino acid increases embryo survival by improving the functional placenta area, but also that increasing the vascularisation of the placenta early in gestation can affect foetal growth later in gestation. The only downside of a strategy that increases embryo survival at the same time is that competition between embryos is increased, which may counteract the benefit of an increased placental functional area.

From more recent publications, it is also evident that arginine influences embryonic and foetal developmental processes, and therefore has effects that go beyond increasing the exchange of nutrients between the sow and her conceptuses. Costa et al. [65] for example, reported that at day 25 of gestation, 1% arginine supplementation increased the embryo weight in line with other studies, and interestingly, increased expression of the IGF-1 gene in the embryos. At day 35 however, embryos from dams supplemented with arginine were smaller. In a different paper by the same authors [66], arginine differentially regulated expression of embryonic genes involved in energy metabolism and in the mTOR pathways at day 25 of gestation, whereas at day 35, these effects were not observed. Collectively, these new findings point to the effects of arginine fed to the dam on embryo development and gene expression in addition to the effects described in earlier work on placental capacity. Moreover, the effects of arginine seem to depend on the stage of gestation.

## 7. Loss of Pregnancy Related to Nutrition

Pregnancy failure under commercial conditions is hard to quantify, since it generally goes unnoticed, and is therefore only diagnosed when sows return to oestrus between three and four weeks after mating or appear non-pregnant when diagnosed using ultrasound. Pregnancy failure probably ranges between 5% and 40%, however, it is obvious that to a certain degree pregnancy was never established. Loss of pregnancy may be initiated as early as day 15 of pregnancy when, in the absence of a sufficient number of embryos, luteolysis occurs. This will most likely be a consequence of poor fertilisation and is not likely to be caused by nutrition. As explained above, luteal tissue develops independent of LH until day 12 of pregnancy, and any nutritional effects affecting LH would not induce luteal failure at this stage. The effects of nutrition, independent of LH, such as those mediated by insulin and IGF-1 described above, may alter the amount of luteal tissue and progesterone secretion before implantation (day 12 to 15). However, these are unlikely to affect the number of embryos present between days 12 and 15, when the initial signal of maternal recognition, oestrogen secretion by the embryos, is critical for the maintenance of pregnancy. Low endogenous progesterone levels at day 12 of gestation have been associated with the return to oestrus at day 21 after mating [67]. There are, however, no reports of nutritionally induced failure of pregnancy at day 15 of gestation.

If anything, a nutrition-related failure of pregnancy is more likely to occur once embryos have started implantation and rely more on the uterine environment and interaction with the endometrium for developmental cues and nutrition. Beyond days 10–12, when the corpora lutea do rely on LH, the nutritional effects on LH may compromise pregnancy, but only when they are severe [1]. Moderate alterations in feed level will affect LH secretion, however, not to the extent that pregnancy failure is likely. In periods of seasonal infertility, however, mild effects on LH may be involved in pregnancy failure. LH secretion may be weak under long days and combined with restricted feeding, this may result in reduced progesterone secretion during implantation. Poor signaling between the uterus and embryos may result in the interruption of pregnancy and the return to oestrus between day 25 and 35, which is typical for seasonal infertility [68]. Suboptimal LH secretion around day 12 of gestation was followed by loss of pregnancy in a recent study by Haen et al. [69]. As opposed to long days, short days, in combination with high plane feeding will stimulate LH secretion and increase the chance of maintaining pregnancy [70]. These observations are reflected by the positive effects of abundant feeding on the pregnancy rates in gilts [71] and sows [46]. The effects of limited feed intake and the season may be aggravated in situations where competition for feed results in such low intakes in individual sows that pregnancy fails. Observations by Kongsted et al. [56] in group-housed sows were mentioned above, however, even with electronic sow feeding, sows that have lower feed intake than allowed in the window from day 10 to 30 of gestation, had a lower pregnancy rate [72,73].

## 8. Conclusions

Research into the role of nutrition in early pregnancy in the past decades, has mainly focused on luteal function and the secretion of progesterone, in relation to the establishment of pregnancy, control of endometrial remodling, and embryo survival. In contrast to concepts developed in the 1980s and 1990s, research from the last few decades suggests that high feed intakes in the first month of gestation are beneficial to embryo development and survival. Before implantation, LH-independent cues, such as IGF-1, stimulate luteal tissue formation and progesterone secretion by the ovaries, contributing to the supply of progesterone to the endometrium directly from the ovaries. It is only during the first three days after ovulation that a high feed intake may possibly reduce systemic progesterone, and since, at this stage, direct transfer from the ovaries may still be low, may also reduce embryo survival. After day 12 of pregnancy, energy intake also affects uterine function through LH-dependent pathways and has been proven to benefit embryo survival. There has been little focus on specific nutrients with nutraceutical effects, however, there are opportunities for future research based on the dynamics of specific nutrients in the uterine fluids in this period. Examples of these are leucine, arginine, glutamine, glucose and fructose, riboflavin, and folic acid. Another area of interest may be the effect of nutrition on factors secreted by the embryos in the period of elongation and implantation, such as PGE, EGFs, and TGFs. After implantation, the effects of nutrition on the vascularisation of the placenta has been proven to affect embryo development and survival, as demonstrated by studies using arginine. This is understandable since in this phase uterine capacity becomes limiting not only for the number of embryos it can support, but also for embryo development.

## Figures and Tables

**Table 1:** Embryo survival and development at day 21 and day 35 of gestation in intact sows and in oviduct ligated sows, demonstrating the effect of uterine space (from Langendijk et al. [24]).

Item	Day 21 Intact Sows	Day 21 Oviduct Ligation *	Day 35 Intact Sows	Day 35 Oviduct Ligation *
Number of sows	15	11	17	12
Ovulations	20.9 ± 1.5 ^a^	11.6 ± 0.8 ^b^	20.3 ± 0.9 ^a^	10.7 ± 0.9 ^b^
Viable embryos, % **	76 ± 5	75 ± 5	59 ± 4 ^a^	77 ± 3 ^b^
Length of implantations, cm	9.9 ± 1.1	11.4 ± 1.2	15.5 ± 1.3 ^x^	19.0 ± 1.2 ^y^
Embryo weight, g	0.22 ± 0.03	0.25 ± 0.05	4.3 ± 0.3 ^x^	4.9 ± 0.2 ^y^

* Oviduct ligated sows had one oviduct ligated, limiting the number of fertilised oocytes entering the uterus to those originating from the patent oviduct. These embryos therefore, had twice the space compared to intact sows. ** Percentage of viable embryos: number of viable embryos present/number of ovulations at patent oviduct. ^a,b^ *p* < 0.05, ^x,y^ *p* < 0.10.

**Table 2:** Effects of feed allowance (high or low) in early pregnancy on the amount of luteal tissue.

Reference	Feed Allowance (High vs. Low *)	Duration of Treatments	Luteal Tissue Mass, g		Stage of Gestation
			High	Low	
[3]	2.4 vs. 1.2 M	d1–25	7.2 ^a^	6.7 ^b^	d35
[7]	+2.5 kg	d1–7	9.5 ^a^	7.7 ^b^	d30
[33]	2.4 vs. 1.2 M	d1–10	8.2	7.9	d10
[26]	2.4 vs. 0.8 M	-	No effect	-	-

* Feed allowance expressed as kg or relative to maintenance requirements (M). ^a,b^ *p* < 0.05.

## References

[1] Langendijk P., Peltoniemi O. (2013). How does nutrition influence luteal function and early embryo survival. Soc. Reprod. Fertil..

[2] Almeida F.R.C.L., Mao J., Novak S., Cosgrove J.R., Foxcroft G.R. (2001). Effects of different patterns of feed restriction and insulin treatment during the luteal phase on reproductive, metabolic, and endocrine parameters in cyclic gilts. J. Anim. Sci..

[3] Athorn R.Z., Stott P.G., Bouwman E.G., Edwards A.C., Blackberry M.A., Martin G.B., Langendijk P. (2013). Feeding level and dietary energy source have no effect on embryo survival in gilts, despite changes in systemic progesterone levels. Anim. Prod. Sci..

[4] Willis H.J., Zak L.J., Foxcroft G.R. (2003). Duration of lactation, endocrine and metabolic state, and fertility of primiparous sows. J. Anim. Sci..

[5] Foxcroft G.R. (1997). Mechanisms mediating nutritional effects on embryonic survival in pigs. J. Reprod. Fertil..

[6] Zak L.J., Williams I.H., Foxcroft G.R., Pluske J.R., Cegielski A.J., Clowes E.J., Aherne F.X. (1998). Feeding lactating primiparous sows to establish three divergent metabolic states: I. Associated endocrine changes and postweaning reproductive performance. J. Anim. Sci..

[7] Gerritsen R., Soede N.M., Laurenssen B., Langendijk P., Dieleman S.J., Hazeleger W., Laurenssen B.F.A., Kemp B. (2008). Feeding level does not affect progesterone levels in intermittently suckled sows with lactational ovulation. Anim. Reprod. Sci..

[8] Steinhauser C.B., Bazer F.W., Burghardt R.C., Johnson G.A. (2017). Expression of progesterone receptor in the porcine uterus and placenta throughout gestation: Correlation with expression of uteroferrin and osteopontin. Domest. Anim. Endocrinol..

[9] Waclawik A., Kaczmarek M.M., Blitekl A., Kaczynski P., Ziecik A.J. (2017). Embryo-maternal dialogue during pregnancy establishment and implantation in the pig. Mol. Reprod. Dev..

[10] Ford S.P., Vonnahme K.A., Wilson M.E. (2002). Uterine capacity in the pig reflects a combination of uterine environment and conceptus genotype effects. J. Anim. Sci..

[11] Anderson L.H., Christenson L.K., Christenson R.K., Ford S.P. (1993). Investigations into the control of litter size in swine: II. Comparisons of morphological and functional embryonic diversity between Chinese and American breeds. J. Anim. Sci..

[12] Dziuk P. (1985). Effect of migration, distribution and spacing of pig embryos on pregnancy and fetal survival. J. Reprod. Fertil..

[13] Pope W.F., Lawyer M.S., First N.L. (1986). Intrauterine migration of the porcine embryo: Coordination of bead migration with estradiol. J. Anim. Sci..

[14] Dziuk P.J. (1968). Effect of number of embryos and terine space on embryonic survival in the pig. J. Anim. Sci..

[15] Geisert R.D., Schmitt R.A.M. (2002). Early embryonic survival in the pig: Can it be improved?. J. Anim. Sci..

[16] Geisert R.D., Ross J.W., Ashworth M.D., White F.J., Johnson G.A., Da Silva U. (2006). Maternal recognition of pregnancy or endocrine disruptor: The two faces of oestrogen during establishment of pregnancy in the pig. Soc. Reprod. Suppl..

[17] Morgan G.L., Geisert R.D., Zavy M.T., Fazleabas A.T. (1987). Development and survival of pig blastocysts after oestrogen administration on day 9 or days 9 and 10 of pregnancy. J. Reprod. Fertil..

[18] Rampacek G.R., Robison O.W., Ulberg L.C. (1975). Uterine Capacity and Progestin Levels in Superinducted Gilts. J. Anim. Sci..

[19] Chen Z.Y., Dziuk P.J. (1993). Influence of initial length of uterus per embryo and gestation stage on prenatal survival, development, and sex ratio in the pig. J. Anim. Sci..

[20] Père M.C., Dourmad J.-Y., Etienne M. (1997). Effect of number of pig embryos in the uterus on their survival and development and on maternal metabolism. J. Anim. Sci..

[21] Town S.C., Putman C.T., Turchinsky N.J., Dixon W.T., Foxcroft G.R. (2004). Number of conceptuses in utero affects porcine fetal muscle development. Reproduction.

[22] Pope C.E., Christenson R.K., Zimmerman-Pope V.A., Day B.N. (1972). Effect of number of embryos on embryonic survival in recipient gilts. J. Anim. Sci..

[23] Webel S.K., Dziuk P.J. (1974). Effect of stage of gestation and uterine space on prenatal survival in the pig. J. Anim. Sci..

[24] Langendijk P., Chen T.Y., Athorn R.Z., Bouwman E.G. (2016). Embryonic survival at day 9, 21 and 35 of pregnancy in intact and unilaterally oviduct ligated multiparous sows. Animal.

[25] Peltoniemi O.A.T., Easton B.G., Love R.J., Klupiec C., Evans G. (1995). Effect of chronic treatment with a GnRH agonist (Goserelin) on LH secretion and early pregnancy in gilts. Anim. Reprod. Sci..

[26] Quesnel H., Pasquier A., Mounier A.-M., Prunier A. (2000). Feed restriction in cyclic gilts: Gonadotrophin-independent effects on follicular growth. Reprod. Nutr. Dev..

[27] Chen T.Y., Stott P., Athorn R.Z., Bouwman E.G., Langendijk P. (2012). Undernutrition during early follicle development has irreversible effects on ovulation rate and embryos. Reprod. Fertil. Dev..

[28] Ashworth C.J., Beattie L., Antipatis C. (1999). Effects of pre- and post-mating nutritional status on hepatic function, progesterone concentration, uterine protein secretion and embryo survival in meishan pigs. Reprod. Fertil. Dev..

[29] Mao J., Zak L.J., Cosgrove J.R., Shostak S., Foxcroft G.R. (1999). Reproductive, metabolic, and endocrine responses to feed restriction and gnrh treatment in primiparous, lactating sows. J. Anim. Sci..

[30] Mao J., Treacy B.K., Almeida F.R.C.L., Novak S., Dixon W.T., Foxcroft G.R. (2001). Feed Restriction and Insulin Treatment Affect Subsequent Luteal Function in the Immediate Postovulatory Period in Pigs: Progesterone Production In Vitro and Messenger Ribonucleic Acid Expression for Key Steroidogenic Enzymes. Biol. Reprod..

[31] Chen T.Y., Stott P., Bouwman E.G., Langendijk P. (2013). Effects of Pre-Weaning Energy Substitutions on Post-Weaning Follicle Development, Steroid Hormones and Subsequent Litter Size in Primiparous Sows. Reprod. Domest. Anim..

[32] Wientjes J.G.M., Soede N.M., van den Brand H., Kemp B. (2011). Nutritionally induced relationships between insulin levels during the weaning-to-ovulation interval and reproductive characteristics in multiparous sows: II. Luteal development, progesterone and conceptus development and uniformity. Reprod. Domest. Anim..

[33] Athorn R.Z., Stott P., Bouwman E.G., Chen T.Y., Kennaway D.J., Langendijk P. (2012). Effect of feeding level on luteal function and progesterone concentration in the vena cava during early pregnancy in gilts. Reprod. Fertil. Dev..

[34] Ferguson E.M., Ashworth C.J., Edwards S.A., Hawkins N., Hepburn N., Hunter M.G. (2003). Effect of different nutritional regimens before ovulation on plasma concentrations of metabolic and reproductive hormones and oocyte maturation in gilts. Reproduction.

[35] Novak S., Treacy B.K., Almeida F.R.C.L., Mao J., Buhi W.C., Dixon W.T., Foxcroft G.R. (2002). Regulation of IGF-I and porcine oviductal secretory protein (pOSP) secretion into the pig oviduct in the peri-ovulatory period, and effects of previous nutrition. Reprod. Nutr. Dev..

[36] Ptak A., Gregoraszczuk E.L., Rzasa J. (2003). Growth hormone and insulin-like growth factor-I action on progesterone secretion by porcine corpora lutea isolated at various periods of the luteal phase. Acta Vet. Hung..

[37] Miller E.A., Ge Z., Hedgpeth V., Gadsby J.E. (2003). Steroidogenic responses of pig corpora lutea to insulin-like growth factor I (IGF-I) throughout the oestrous cycle. Reproduction.

[38] Ptak A., Kajta M., Gregoraszczuk E.L. (2004). Effect of growth hormone and insulin-like growth factor-I on spontaneous apoptosis in cultured luteal cells collected from early, mature, and regressing porcine corpora lutea. Anim. Reprod. Sci..

[39] Langendijk P., van den Brand H., Gerritsen R., Quesnel H., Soede N.M., Kemp B. (2008). Porcine luteal function in relation to IGF-1 levels following ovulation during lactation or after weaning. Reprod. Domest. Anim..

[40] Prime G.R., Symonds H.W. (1993). Influence of plane of nutrition on portal blood flow and the metabolic clearance rate of progesterone in ovariectomised gilts. J. Agric. Sci..

[41] Jindal R., Cosgrove J.R., Aherne F.X., Foxcroft G.R. (1996). Effect of nutrition on embryonal mortality in gilts: Association with progesterone. J. Anim. Sci..

[42] Langendijk P., Farmer C. (2015). Early gestation feeding and management for optimal reproductive performance. The Gestating and Lactating Sow.

[43] Leal D.F., Muroa B.B.D., Nichia M., Almond G.W., Viana C.H.C., Viotid G., Carnevale R.F., Garbossa C.A.P. (2019). Effects of post-insemination energy content of feed on embryonic survival in pigs: A systematic review. Anim. Reprod. Sci..

[44] Athorn R.Z., Stott P., Bouwman E.G., Ashman R., O’Leary S., Nottle M., Langendijk P. (2011). Direct ovarian-uterine transfer of progesterone increases embryo survival in gilts. Reprod. Fertil. Dev..

[45] Stefanczyk-Krzymowska S., Grzegorzewski W., Wasowska B., Skipor J., Krzymowski T. (1998). Local increase of ovarian steroid hormone concentration in blood supplying the oviduct and uterus during early pregnancy of sows. Theriogenology.

[46] Virolainen J.V., Love R.J., Tast A., Peltoniemi O.A. (2005). Plasma progesterone concentration depends on sampling site in pigs. Anim. Reprod. Sci..

[47] Bazer F.W., Kim J., Song G., Ka H., Wu G., Johnson G.A., Vallet J.L., Rodrigues-Martinez H. (2013). Roles of selected nutrients in development of the porcine conceptus during pregnancy. Control of Pig Reproduction IX, Proceedings of the International Conference on Pig Reproduction, Olsztyn, Poland, 9–13 June 2013.

[48] Lindemann M.D. (1993). Supplemental folic acid: A requirement for optimizing swine reproduction. J. Anim. Sci..

[49] Gaudré D., Quiniou N. (2009). What mineral and vitamin levels to recommend in swine diets?. Rev. Bras. Zootec..

[50] Matte J.J., Farmer C., Girard C.L., Laforest J.-P. (1996). Dietary folic acid, uterine function and early embryonic development in sows. Can. J. Anim. Sci..

[51] Tremblay G.F., Matte J.J., Dufour J.J., Brisson G.J. (1989). Days of gestation after folic acid addition to a swine diet survival rate and development of fetuses during the first 30 days. J. Anim. Sci..

[52] Harper A.F., Knight J.W., Kokue E., Usry J.L. (2003). Plasma reduced folates, reproductive performance, and conceptus development in sows in response to supplementation with oxidized and reduced sources of folic acid. J. Anim. Sci..

[53] Guay F., Matte J.J., Girard C.L., Palin M.-F., Giguère A., Laforest J.-P. (2002). Effect of folic acid and glycine supplementation on embryo development and folate metabolism during early pregnancy in pigs. J. Anim. Sci..

[54] Bazer F.W. (1991). Use of supplemental dietary riboflavin to increase fertility and/or prolificacy in animals. U.S. Patent.

[55] Langendijk P., Bouwman E.G., Chen T.Y., Koopmanschap R.E., Soede N.M. (2017). Effects of temporary undernutrition on ovarian progesterone secretion and corpora lutea morphometrics during early gestation in gilts. Reprod. Fertil. Dev..

[56] Kongsted A.G. (2004). Reproduction Performances and Conditions of Group-Housed Non-Lactating Sows. Ph.D. Thesis.

[57] Athorn R.Z., Stott P., Langendijk P., Van Barneveld R.J. (2011). Feeding level and dietary energy source during early pregnancy in first parity sows: Effects on pregnancy and litter size. Proceedings of the Australasian Pig Science Association, Adelaide, Australia, 27–30 November 2011.

[58] Langendijk P., Athorn R.Z., Stott P., Van Barneveld R.J. (2011). Feeding level and dietary fibre content during early pregnancy in gilts and pregnancy rate and litter size. Proceedings of the Australasian Pig Science Association: Adelaide, Australia, 27–30 November 2011.

[59] Jensen M.B., Pedersen L.J., Theil P.K., Yde C.C., Knudsen K.E.B. (2012). Feeding motivation and plasma metabolites in pregnant sows fed diets rich in dietary fiber either once or twice daily. J. Anim. Sci..

[60] Knudsen K.E.B., Henry J., Canibe N. (2000). Quantification of the absorption of nutrients derived from carbohydrate assimilation: Model experiment with catheterised pigs fed on wheat- or oat-based rolls. Brit. J. Nutr..

[61] Novak S., Paradis F., Patterson J.L., Pasternak J.A., Oxtoby K., Moore H.S., Hahn M., Dyck M.K., Dixon W.T., Foxcroft G.R. (2012). Temporal candidate gene expression in the sow placenta and embryo during early gestation and effect of maternal Progenos supplementation on embryonic and placental development. Reprod. Fertil. Dev..

[62] Hazeleger W., Smits C., Kemp B., Wiseman J., Kemp B., Varley M. (2007). Influence of nutritional factors on placental growth and piglet imprinting. Paradigms in Pig Science.

[63] Ramaekers P., Kemp B., van der Lende T. (2006). Progenos in sows increases number of piglets born. J. Anim. Sci..

[64] Wu G., Bazer F.W., Burghardt R.C., Johnson G.A., Kim S.W., Li X.L., Satterfield M.C., Spencer T.E. (2010). Impacts of amino acid nutrition on pregnancy outcome in pigs: Mechanisms and implications for swine production. J. Anim. Sci..

[65] Costa K.A., Saraiva A., Guimarães J.D., Botelho D., Marques D., Machado-Neves M., Barbosa L.M.R., Villadiego F.A.C., Veroneze R., de Oliveira L.F. (2019). Dietary L-arginine supplementation during early gestation of gilts affects conceptuses development. Theriogenology.

[66] Garcia I.S., Teixeira S.A., Costa K.A., Marques D.B.D., de Amorim Rodrigues G., Costa T.C., Guimarães J.D., Otto P.I., Saraiva A., Ibelli A.M.G. (2020). L-Arginine supplementation of gilts during early gestation modulates energy sensitive pathways in pig conceptuses. Mol. Reprod. Dev..

[67] Langendijk P., Dieleman S.J., Gerritsen R., Hazeleger W., Mainsant M.-L., Soede N.M., Kemp B. (2007). Pulsatile release of luteinising hormone during the luteal phase in lactating and weaned sows. Reprod. Fertil. Dev..

[68] Tast A., Peltoniemi O., Virolainen J., Love R. (2002). Early disruption of pregnancy as a manifestation of seasonal infertility in pigs. Anim. Reprod. Sci..

[69] Haen S.M., Heinonen M., Bjorkmann S., Soede N.M., Peltoniemi O.A.T. (2020). Progesterone and luteinizing hormone secretion patterns in early pregnant gilts. Reprod. Dom. Anim..

[70] Peltoniemi O.A., Virolainen J.V. (2006). Seasonality of reproduction in gilts and sows. Soc. Reprod. Fertil. Suppl..

[71] Virolainen J.V., Tast A., Sorsa A., Love R.J., Peltoniemi O.A.T. (2004). Changes in feeding level during early pregnancy affect fertility in gilts. Anim. Reprod. Sci..

[72] Athorn R.Z., Sawyer K.S., Collins C.L., Luxford B.G., Pluske J., Pluske J. (2013). High growth rates during early pregnancy positively affect farrowing rate in parity one and two sows. Manipulating Pig Production XIV.

[73] Sawyer K.S., Athorn R.Z., Collins C.L., Luxford B.G., Pluske J., Pluske J. (2013). Increasing feed intake in early gestation improves farrowing rate in first and second parity sows. Manipulating Pig Production XIV.

